# IS THERE A RELATIONSHIP BETWEEN PAIN AND FEAR OF FALLING IN OLDER ADULTS LIVING IN LONG-TERM CARE FACILITIES?

**DOI:** 10.1093/geroni/igz038.2635

**Published:** 2019-11-08

**Authors:** Edgar R Vieira, Diego Tavares, Particia Nobrega, Alvaro Maciel

**Affiliations:** 1 Department of Physical Therapy, Florida International University, Miami, Florida, United States; 2 Graduate Program in Physical Therapy, Federal University of Rio Grande do Norte, Natal, Rio Grande do Norte, Brazil

## Abstract

Fear of falling is common in older adults and it is associated with multiple factors such as gait and balance issues, difficulties in activities of daily living, visual impairment, and frailty. Unfortunately, fear of falls increases the risk of falls as opposed to protect from falls. Pain can impair mobility, affects activities of daily living, and may also be associated with fear of falling but no studies have evaluated this potential association. The objectives of this study were to evaluate if there was an association between pain and fear of falling in older adults living in long-term care facilities. One hundred and eight older adults living in long-term care facilities participated in the study. The mean age was 79±7 years, and 65% of the participants were women. The participants completed the Geriatric Pain Measure (GPM) questionnaire for multidimensional pain assessment (scores range from 0 to 42), and the Falls Efficacy Scale International (FES-I) for fear of falling assessment (scores range from 16 to 64). The data was analyzed using multiple linear regression. Forty-five percent of the participants had chronic pain (≥3 months) and 18% had acute pain (<3 months). Pain scores were 29±31. Pain was associated with an increase of 3 to 7 points (out of 64 max) in the FES-I. The prevalence of pain in long-term care residents was high, and pain was associated with increased fear of falling.

